# Supplementary material for: Plant DNA polymerases α and δ mediate replication of geminiviruses
Source: Nat Commun. 2021 May 13;12:2780. doi: 10.1038/s41467-021-23013-2 (PMC8119979; doi:10.1038/s41467-021-23013-2)
Supplement: Supplementary file 1 — Supplementary Information [file 41467_2021_23013_MOESM1_ESM.pdf]

SUPPLEMENTARY INFORMATION

Plant DNA polymerases α and δ mediate replication of geminiviruses

Mengshi Wu, Hua Wei, Huang Tan, Shaojun Pan, Qi Liu, Eduardo R Bejarano, Rosa Lozano-Durán

This file contains:

Supplementary figures 1 to 9
Supplementary tables 1 to 3
Supplementary references

SUPPLEMENTARY FIGURES

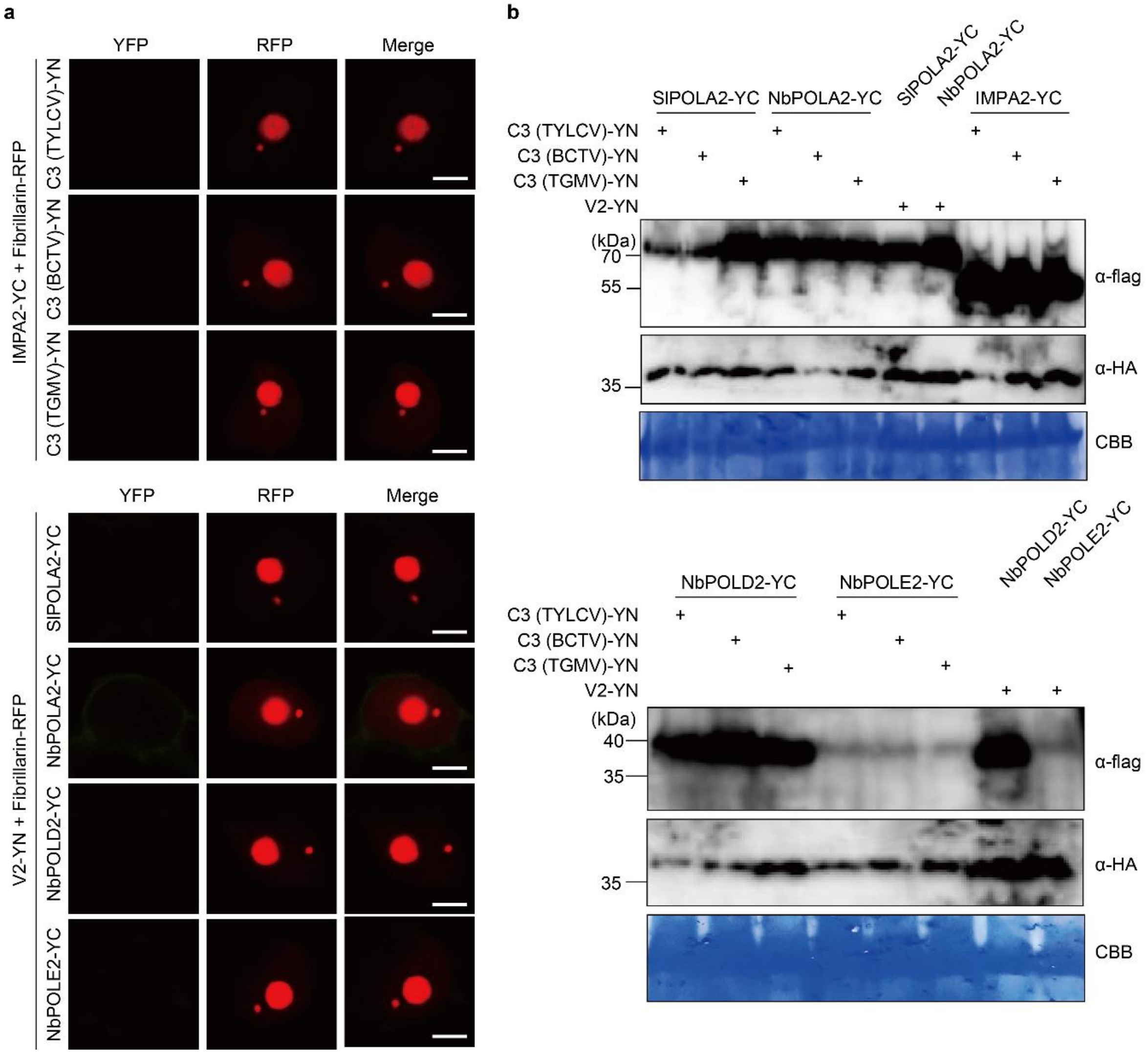

Supplementary figure 1. Negative controls for the BiFC experiments in Figures 1d and 2a, b. IMPA2 from *Arabidopsis thaliana* and V2 from TYLCV are used as negative controls (a). Scale bar: 5 µm. Accumulation of the proteins in (a) is shown in (b). The predicted proteins sizes are as follows: SlPOLA2-YC: ~76 kDa; NbPOLA2-YC: ~76 kDa; NbPOLD2-YC: ~28 kDa; NbPOLE2-YC: ~44 kDa; IMPA2-YC: ~66 kDa; C3 (TYLCV)-YN: ~35 kDa; C3 (BCTV)-YN: ~36 kDa; C3 (TGMV)-YN: ~35 kDa; V2-YN: ~33 kDa. YC fusions contain a Flag tag; YN fusions contain an HA tag. CBB: Coomassie brilliant blue. Anti-Flag (Abmart, M20008) and anti-HA (Roche, Cat. No. 11583816001) were used to detect the Flag-tagged or HA-tagged proteins separately. Full blots and membrane can be found in the Source data file. This experiment was repeated three times with similar results.

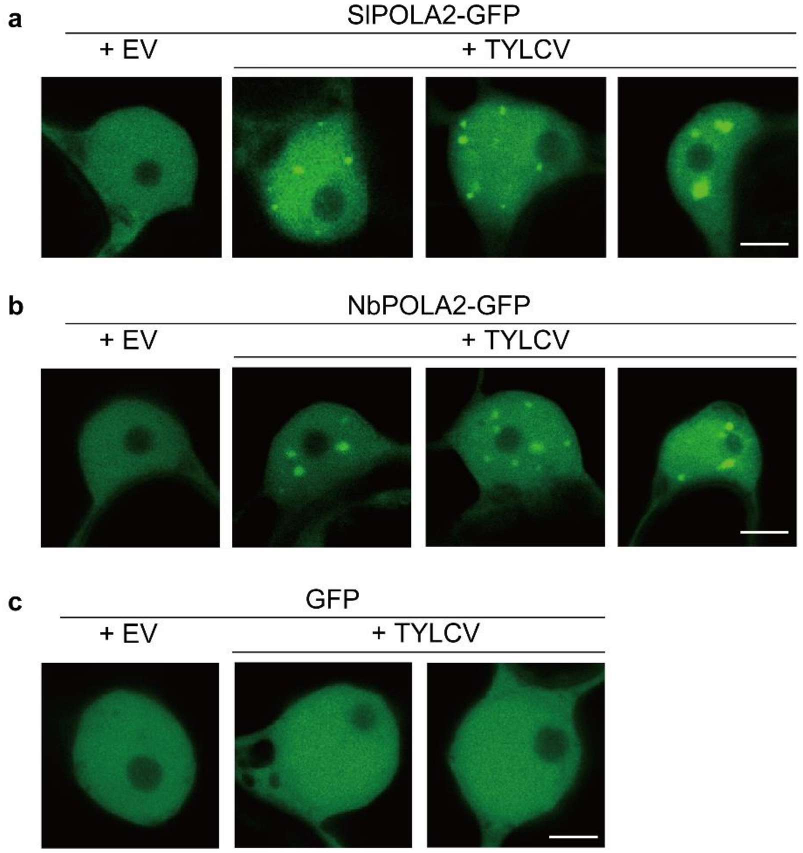

Supplementary figure 2. Nuclear distribution of transiently expressed SlPOLA2-GFP (a), NbPOLA2-GFP (b), and free GFP (c) in the absence (empty vector, EV) or presence of TYLCV in *N. benthamiana*. *Agrobacterium tumefaciens* clones containing the corresponding binary vectors were mixed at 1:1 ratio; images were taken at 2 days post-inoculation. Scale bar: 5 µm. Additional images can be found in Figure 1e. This experiment was repeated more than three times with similar results.

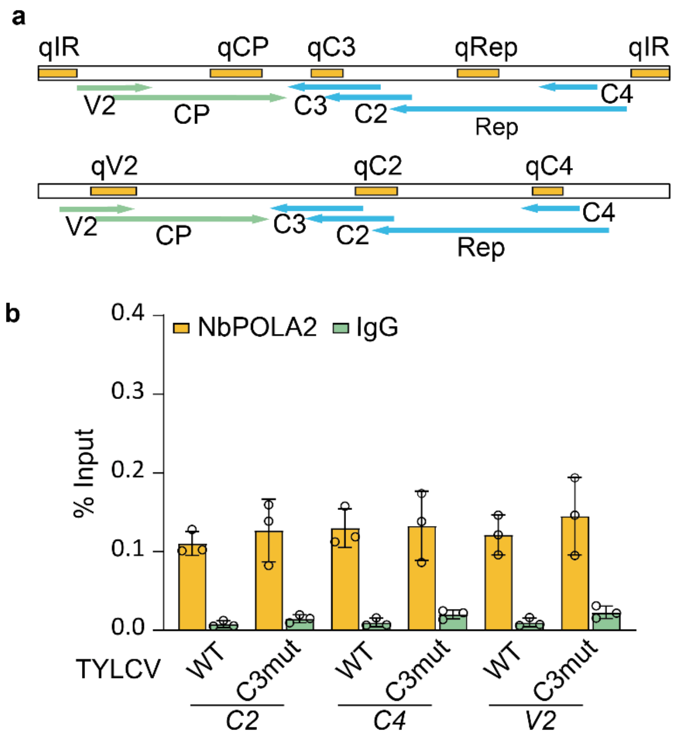

Supplementary figure 3. Genomic location of the viral regions amplified in the ChIP assays (in yellow) (a), and binding of NbPOLA2 to the C2, C4, and V2 regions (b). In (a), viral genes are depicted as arrows; genes in the viral strand are coloured in light green; genes in the complementary strand are coloured in blue. In (b), data are the mean of 3 independent biological replicates; error bars represent SD. This experiment was repeated twice with similar results. The original data from all replicates can be found in the Source data file.

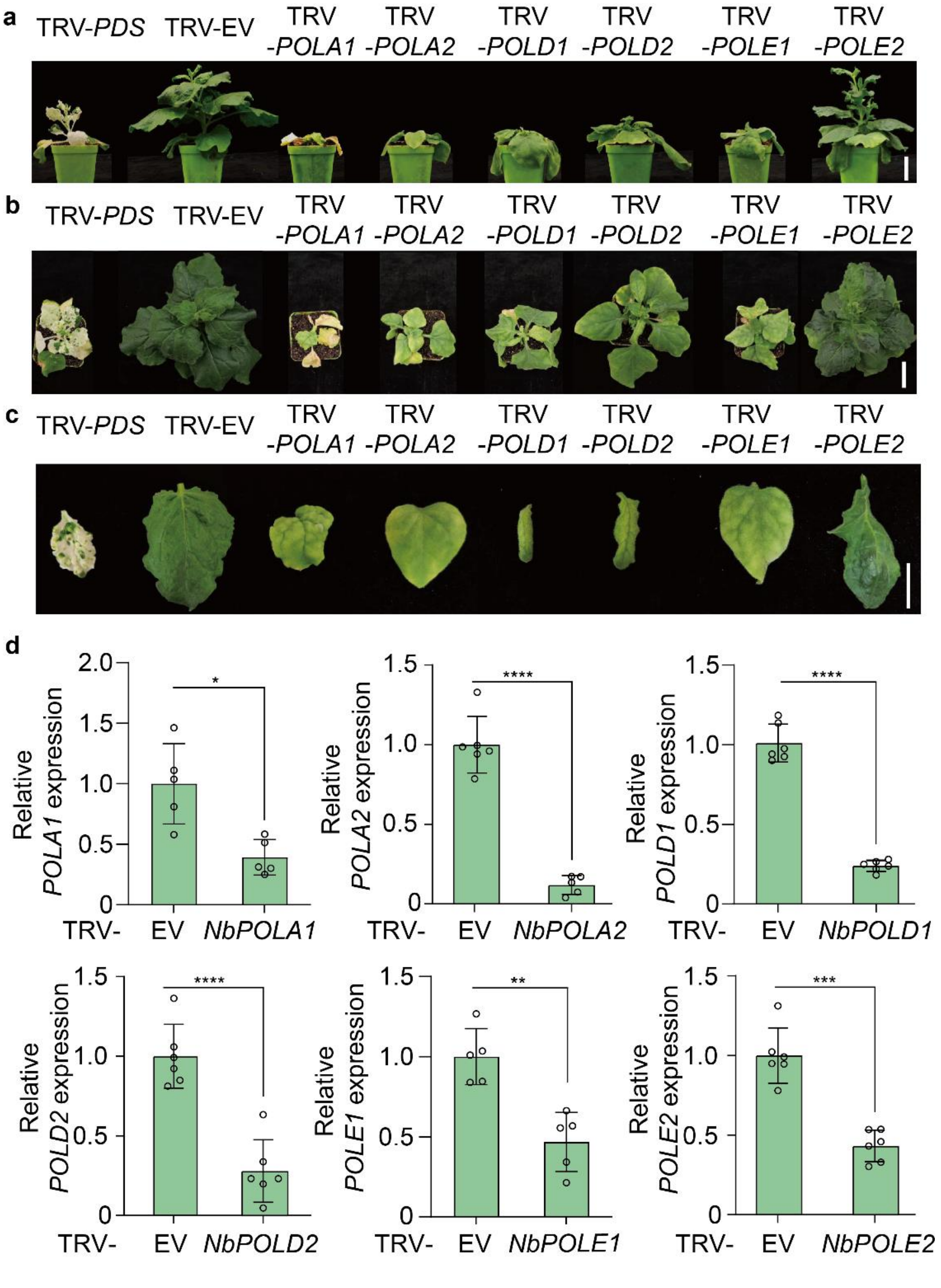

Supplementary figure 4. Developmental phenotypes of *NbPOLA1-*, *NbPOLA2-*, *NbPOLD1-*, *NbPOLD2-*, *NbPOLE1-*, and *NbPOLE2*-silenced (inoculated with TRV-NbPOLA1, TRV-NbPOLA2, TRV-NbPOLD1, TRV-NbPOLD2, TRV-NbPOLE1, and TRV-NbPOLE2, respectively) *N. benthamiana* plants (a-c) and silencing efficiency (d). Lateral

(a) and top (b) views are shown; individual leaves are shown in (c). TRV empty vector (TRV-EV) and TRV-NbPDS are included as controls. Images were taken at 3 weeks post-inoculation. Scale bar: 5 cm. d. *NbPOLA1*, *NbPOLA2*, *NbPOLD1*, *NbPOLD2*, *NbPOLE1*, and *NbPOLE2* transcript accumulation in silenced and control plants, measured by qRT-PCR. *NbActin* was used as reference gene. Values are presented relative to those in the TRV-EV plants. Data are the mean of 5 (*NbPOLA1* and *NbPOLE1*) or 6 (*NbPOLA2*, *NbPOLD1, NbPOLD2* and *NbPOLE2*) independent biological replicates; error bars represent SD. Samples were taken at 2 weeks post-inoculation. Asterisks indicate a statistically significant difference according to two-sided Student's t-test (****, P<0.0001; ***, P<0.001; **, P<0.01; *, P<0.05). This experiment was repeated more than three times with similar results. The original data from all replicates can be found in the Source data file.

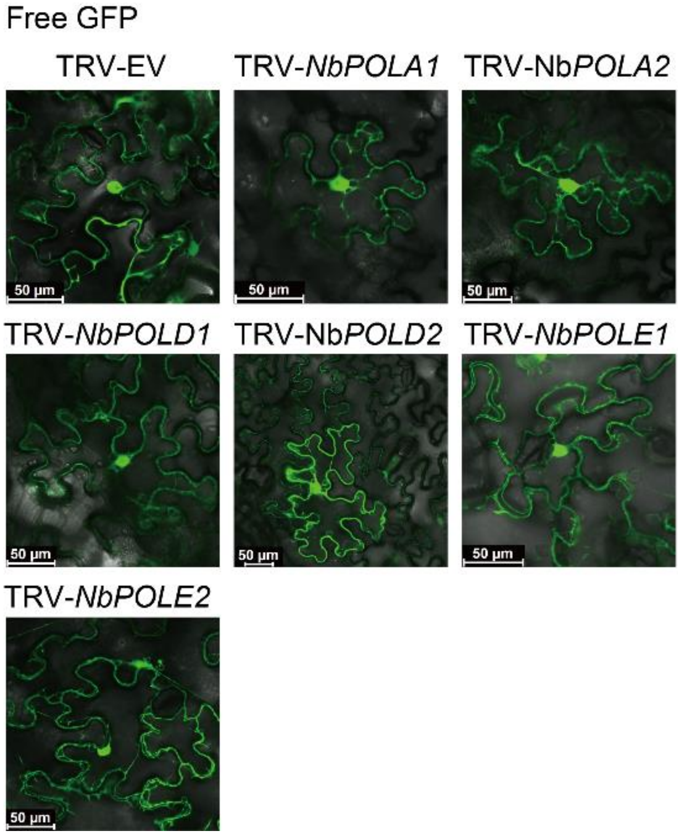

Supplementary figure 5. Silencing of *NbPOLA1*, *NbPOLA2*, *NbPOLD1*, *NbPOLD2*, *NbPOLE1*, or *NbPOLE2* does not affect *Agrobacterium tumefaciens*-mediated transient expression of free GFP in *N. benthamiana*. TRV-EV: empty vector control. Scale bar: 50 µm. Images were taken under the confocal microscope 2 days after transient transformation of plants inoculated with the TRV constructs 3 weeks earlier (see Supplementary figure 4). This experiment was repeated three times with similar results.

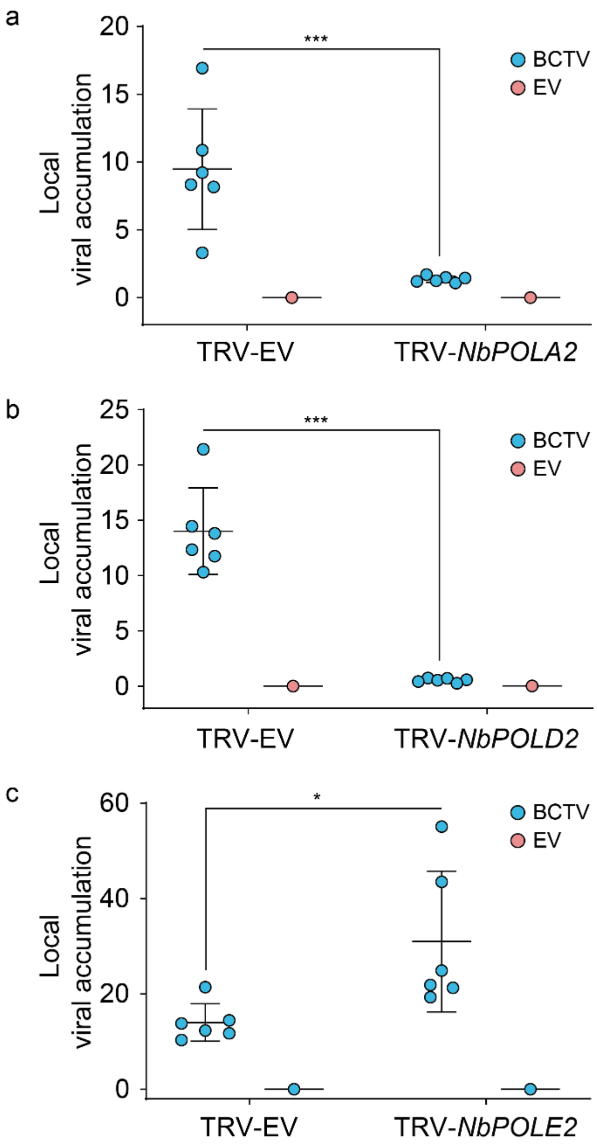

Supplementary figure 6. Viral accumulation in local (3 days post-inoculation) BCTV infections in *NbPOLA2*-, *NbPOLD2*-, *NbPOLE2*-silenced (inoculated with TRV-NbPOLA2, TRV-NbPOLD2, and TRV-NbPOLE2, respectively) or control (TRV-EV) *N. benthamiana* plants measured by qPCR. Plants inoculated with the empty vector (EV) are used as negative control. Data are the mean of 6 independent biological replicates; error bars represent SD. The 25S ribosomal DNA interspacer (*ITS*) was used as reference gene; values are represented relative to *ITS*. These experiments were repeated three times with similar results. Asterisks indicate a statistically significant difference

according to two-sided Student’s t-test (***, $P<0.001$; *, $P<0.05$). The original data from all replicates can be found in the Source data file.

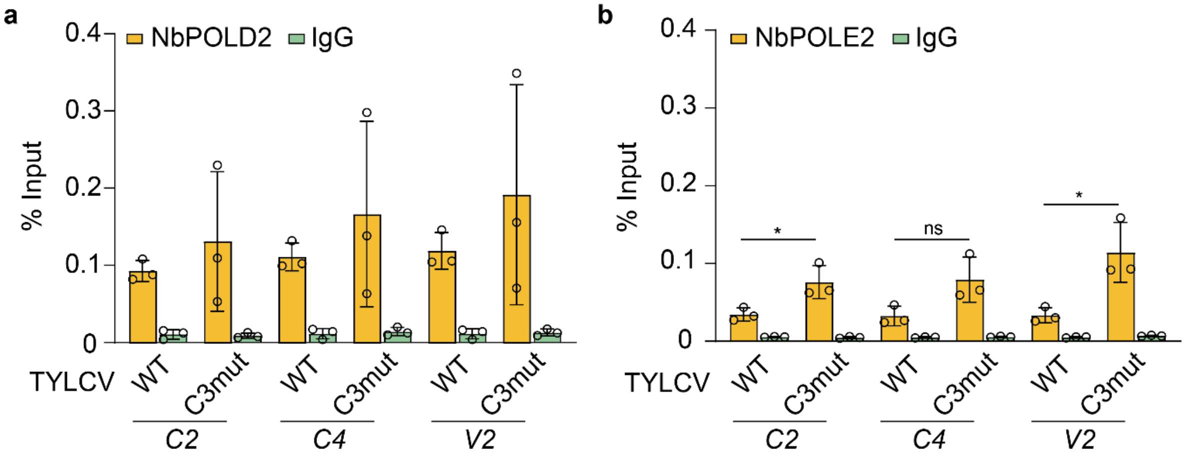

Supplementary figure 7. Binding of NbPOLD2 (a) and NbPOLE2 (b) to the C2, C4, and V2 regions of the TYLCV genome. Data are the mean of 3 independent biological replicates; error bars represent SD. These experiments were repeated twice with similar results. The position of the amplified sequences in the viral genome is shown in Supplementary figure 3a. Asterisks indicate a statistically significant difference according to two-sided Student's t-test (*, P<0.05). ns: non-significant (P>0.05). The original data from all replicates can be found in the Source data file.

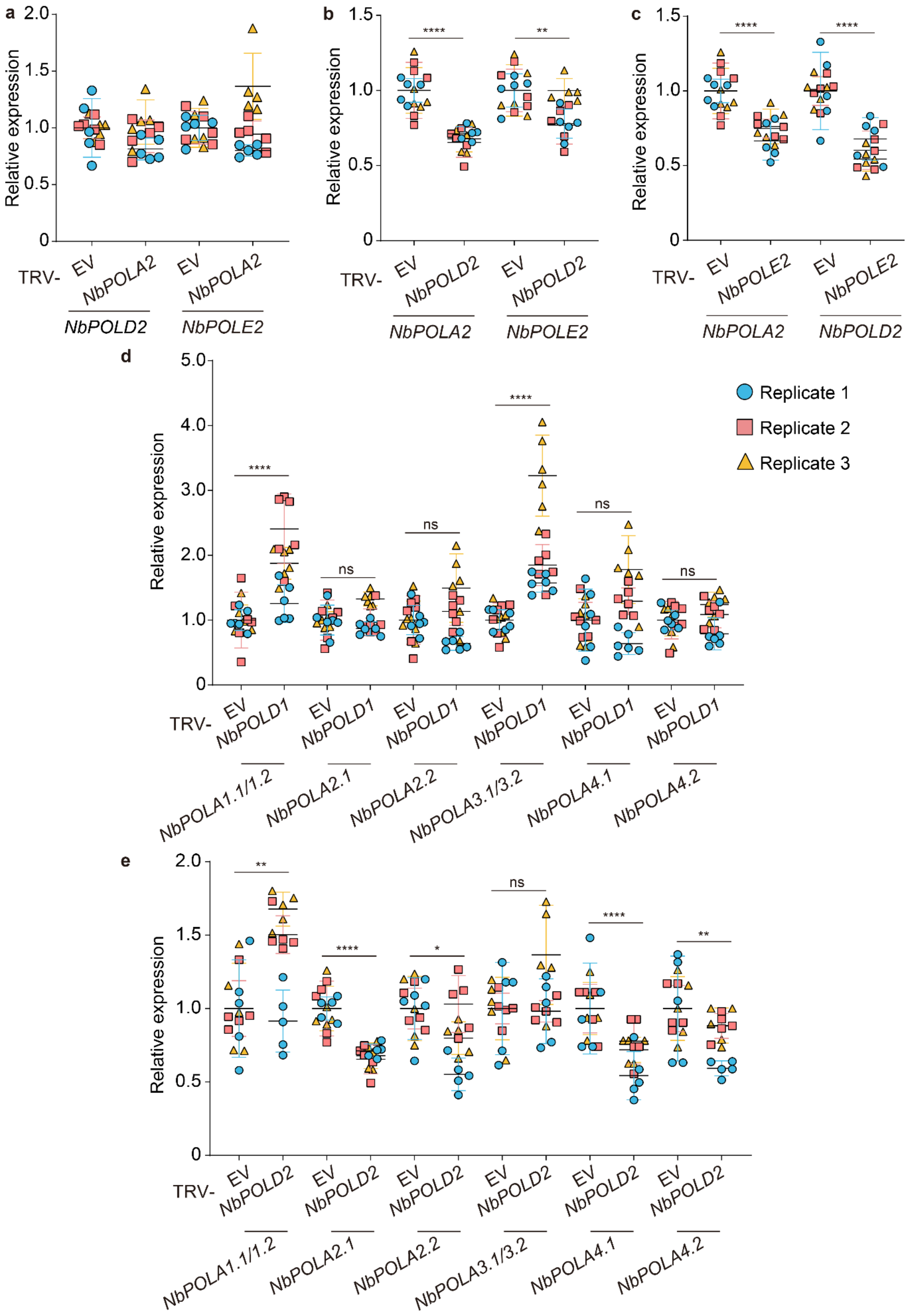
a
b
c
d
e
Relative expression
TRV-
EV
NbPOLA2
NbPOLD2
NbPOLE2
NbPOLD1
NbPOLA1.1/1.2
NbPOLA2.1
NbPOLA2.2
NbPOLA3.1/3.2
NbPOLA4.1
NbPOLA4.2
Replicate 1
Replicate 2
Replicate 3
ns
****
**
*

Supplementary figure 8. Transcript accumulation of different DNA polymerase subunits in silenced plants. a. *NbPOLD2* and *NbPOLE2* transcript accumulation in *NbPOLA2*-silenced plants. b. *NbPOLA2* and *NbPOLE2* transcript accumulation in *NbPOLD2*-silenced plants. c. *NbPOLA2* and *NbPOLD2* transcript accumulation in *NbPOLE2*-silenced plants. d, e. Accumulation of transcripts encoding each subunit of DNA polymerase α in NbPOLD1- (d) or NbPOLD2-silenced plants (e). Transcript accumulation was measured by qRT-PCR. *NbActin* was used as reference gene. Values of three independent biological replicates are shown. Data are the mean of 5 (for *NbPOLA2*-, *NbPOLD2*- and *NbPOLE2*-silenced plants) or 6 (for *NbPOLD1*-silenced plants) independent biological replicates; error bars represent SD. Asterisks indicate a statistically significant difference according to two-sided Student's t-test (****, $P<0.0001$; **. $P<0.01$; *, $P<0.05$). The original data from all replicates can be found in the Source data file.

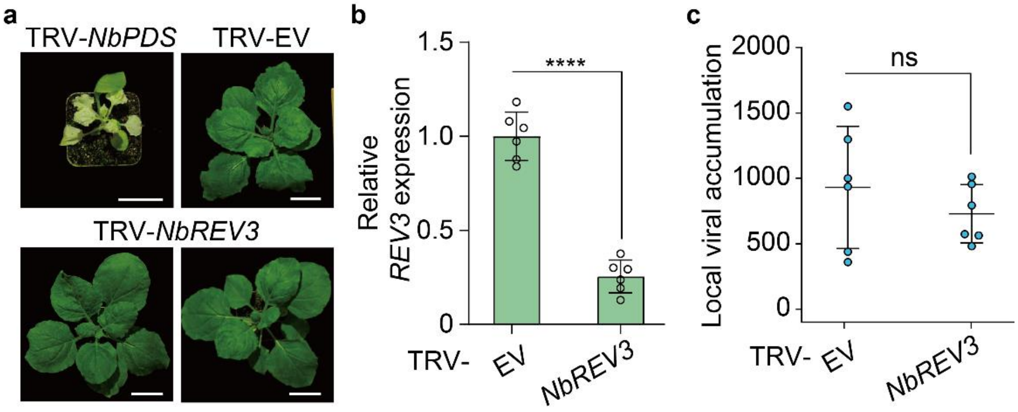

Supplementary figure 9. Silencing of the DNA polymerase ζ subunit REV3 does not affect viral accumulation in local TYLCV infection. a. Developmental phenotype of *NbREV3*-silenced plants (TRV-NbREV3). TRV-NbPDS and TRV-EV are used as positive and negative control, respectively. Scale bar: 5 cm. b. Silencing efficiency of TRV-NbREV3*.* Data are the mean of 6 independent biological replicates; error bars represent SD. Asterisks indicate a statistically significant difference according to two-sided Student's t-test (****, P<0.0001). c. TYLCV accumulation in local infection assays (3 days post-inoculation). Error bars represent SD with n=6 independent biological replicates. The 25S ribosomal DNA interspacer (*ITS*) was used as reference; values are represented relative to *ITS*. Ns indicates no statistically significant difference according to two-sided Student's t-test (P>0.05). These experiments were repeated three times with similar results. The original data from all replicates can be found in the Source data file.

	EV_1	EV_2	EV_3	TYLCV_1	TYLCV_2	TYLCV_3
POLA1						
Niben101Scf01802g02001 (*POLA1.1*)	188	216	310	21	19	13
**Niben101Scf04003g04002** (*POLA1.2*)	**256**	**257**	**409**	**36**	**22**	**58**
POLA2						
**Niben101Scf18951g00009** (*POLA2.1*)	**182**	**169**	**233**	**200**	**146**	**152**
Niben101Scf01073g04022 (*POLA2.2*)	89	51	83	31	19	19
POLA3						
Niben101Scf01950g03015 (*POLA3.1*)	219	108	206	127	100	92
Niben101Scf00366g01013 (*POLA3.2*)	498	415	589	308	336	315
POLA4						
Niben101Scf18347g00003 (*POLA4.1*)	45	58	86	17	8	4
Niben101Scf13695g01002 (*POLA4.2*)	65	66	119	25	24	19
Niben101Scf04053g02011	101	110	148	7	17	13
POLD1						
**Niben101Scf02230g03027**	**363**	**258**	**421**	**393**	**245**	**323**
Niben101Scf00215g00022	74	63	68	62	30	56
Niben101Scf10041g00012	85	53	84	77	35	51
POLD2						
Niben101Scf02793g17003	50	42	42	45	34	26
Niben101Scf02793g17022	66	43	65	44	56	38
**Niben101Scf07121g02003**	**311**	**264**	**362**	**442**	**347**	**341**
Niben101Scf14412g00012	0	0	0	0	0	0
Niben101Scf01230g04020	0	0	0	0	0	0
Niben101Scf09867g02025	0	0	0	0	0	0
Niben101Scf06725g02018	0	0	0	0	0	0
Niben101Scf02449g02012	0	0	0	0	0	0
Niben101Scf08523g00014	0	0	0	0	0	0
Niben101Scf07590g05005	0	0	0	0	0	1
POLD3						
Niben101Scf00160g10002	179	125	185	157	193	142
POLD4						
Niben101Scf02887g01014	6	11	13	2	2	3
Niben101Scf00171g00003	65	51	79	72	71	70
Niben101Scf08228g04010	133	99	117	128	162	154
POLE1						

**Niben101Scf11937g01021**	**105 6**	**610**	**119 2**	**937**	**383**	**783**
POLE2						
**Niben101Scf08137g03002**	**550**	**421**	**717**	**187**	**231**	**207**
Niben101Scf00887g00009	138	103	152	68	47	46
Niben101Scf02174g02008	3	0	5	15	1	2
REV3						
**Niben101Scf10144g01025**	**25**	**20**	**25**	**65**	**38**	**66**

Supplementary table 1. Orthologues of the *Arabidopsis thaliana* genes encoding the subunits of DNA polymerases α (POLA), δ (POLD), ε (POLE), and *REV3* in *N. benthamiana*, and their expression in TYLCV-locally infected samples and the corresponding controls. Data (in reads per million) are from Wu *et al*., 2019. Three independent biological replicates (1-3) are shown. EV: empty vector. Genes selected for further experiments are indicated in red.

PLASMIDS		
**Expression cassette/virus**	**Source**	**Vector**
TYLCV-WT	Rosas-Diaz et al., 2018	pGWB501
TYLCV-C3mut	This paper	pGWB501
pBIN1.2 (BCTV)	Briddon et al., 1989	-
35S:C3-GFP	Wang et al., 2017a	pGWB5
35S:GFP-C3	Wang et al., 2017a	pGWB6
35S:SlPOLA2-RFP	This paper	pGWB554
35S:SlPOLA2-GFP	This paper	pGWB505
35S:NbPOLA2-RFP	This paper	pGWB554
35S:NbPOLA2-GFP	This paper	pGWB505
35S:NbPOLD2-RFP	This paper	pGWB554
35S:NbPOLD2-GFP	This paper	pGWB505
35S:NbPOLE2-RFP	This paper	pGWB554
35S:NbPOLE2-GFP	This paper	pGWB505
TRV2:NbPOLA2-1	This paper	pTRV2
TRV2:NbPOLA2-2	This paper	pTRV2
TRV2:NbPOLD2-1	This paper	pTRV2
TRV2:NbPOLD2-2	This paper	pTRV2
TRV2:NbPOLE2-1	This paper	pTRV2
TRV2:NbPOLE2-2	This paper	pTRV2
TRV2:NbPOLA1	This paper	pTRV2
TRV2:NbPOLD1	This paper	pTRV2
TRV2:NbPOLE1	This paper	pTRV2
TRV2:NbREV3	This paper	pTRV2
35S:C3-TYLCV-YN	This paper	pGTQL1211YN
35S:C3-BCTV-YN	This paper	pGTQL1211YN
35S:C3-TGMV-YN	This paper	pGTQL1211YN
35S:SlPOLA2-YC	This paper	pGTQL1221YC
35S:NbPOLA2-YC	This paper	pGTQL1221YC
35S:NbPOLD2-YC	This paper	pGTQL1221YC
35S:NbPOLE2-YC	This paper	pGTQL1221YC
AD-SlPOLA2	This paper	pGADT7
BD-C3 (TYLCV)	This paper	pGBKT7

Supplementary table 2. Plasmids and constructs used in this work.

AMPLIFICATION TARGET	SOURCE	SEQUENCE 5' ~ 3'
**Oligonucleotides to clone in pENTR™/D-TOPO® (F: CACC) and the pDONR™/Zeo (F: GGGGACAAGTTTGTACAAAAAAGCAGGCTNN, R: GGGGACCACTTTGTACAAGAAAGCTGGGTN)**		
TOPO-C3 (TYLCV) (with stop codon)	This paper	F: CACCATGGATTCACGCACAG<br>R: TTAATAAAATTTATATT
TOPO-C3 (TYLCV) (without stop codon)	Medina-Puche et al., 2020	F: CACCATGGATTCACGCACAG<br>R: ATAAAATTTATATTTTATATC
TOPO-C3 (BCTV) (without stop codon)	This paper	F: CACCATGAATGTAATAGAGGA<br>R: GTACAAGTTCATTGCAACAC
TOPO-SlPOLA2 (without stop codon)	This paper	F: CACCATGGAAGAGGAAATCAAAGC<br>R: TATACGAAGGACTGAAGCAC
pDONR/Zeo-NbPOLA2 (without stop codon)	This paper	F:GGGGACAAGTTTGTACAAAAAAGCAGGCTTCATGGAAGAGCAAATCAAAGCTGA<br>R:GGGGACCACTTTGTACAAGAAAGCTGGGTCTATACGAATAACTGAAGCACTTGACAAATCAC
pDONR/Zeo-NbPOLD2 (without stop codon)	This paper	F:GGGGACAAGTTTGTACAAAAAAGCAGGCTTCATGAGTTCAGAATTTGATTTTCTCCT<br>R:GGGGACCACTTTGTACAAGAAAGCTGGGTCTGAGTGGATTTGAGTAGCAAAGCTG
pDONR/Zeo-NbPOLE2 (without stop codon)	This paper	F:GGGGACAAGTTTGTACAAAAAAGCAGGCTTCATGCTGTGTATCCATCACCTGCAGTAC<br>R:GGGGACCACTTTGTACAAGAAAGCTGGGTCCAATGCTGAGAGTTCTACTTCCTGA
**Oligonucleotides to clone in pTRV2**		
TRV2:NbPOLA2-1	This paper	F: ATCGGAATTCAGGAGAACCCCAATGATG<br>R: ATCGGAGCTCCGCTGCTTTGCAGTAAAA
TRV2:NbPOLA2-2	This paper	F: ATCGGAATTCATGAGGTGAAAGTGGCTTGC<br>R: ATCGGAGCTCAAAAGCGTGAAGCACTTGA
TRV2:NbPOLD2-1	This paper	F: ATCGGAATTCTCCAGATTGCAGCAAGTATAC<br>R: ATCGGAGCTCCACCACTGCCACTCCAGTATCTG
TRV2:NbPOLD2-2	This paper	F: ATCGGAATTCCGCCCTGCCACAGCAGCCTCTC<br>R: ATCGGAGCTCCTGAGTCTAATGGATATCTTTTGC
TRV2:NbPOLE2-1	This paper	F: ATCGGAATTCATGCTGTGTATCCATCACCTG<br>R: ATCGGAGCTCCTTGCCTGAGACTTGAAATTGA

TRV2:NbPOLE2-2	This paper	F: ATCGGAATTCAGAAGACTTTGGGAAATCTG<br>R: ATCGGAGCTCGCAGAGATGGCTCTGATGTAT
TRV2:NbPOLA1	This paper	F: ATCGGAATTCTAGAAGAGTCTCGAATCTCT<br>R: ATCGGAGCTCTCCTCTTGATCAGGAACAG
TRV2:NbPOLD1	This paper	F: CGGAATTCCGGGATCTGAGCAAGGAAC<br>R: CGGGATCCCGAAATCTGCTTTTGCTCTTTTG
TRV2:NbPOLE1	This paper	F: ATCGGAATTCATGAACGGCGGTGGAGATA<br>R: ATCGGAGCTCTTTATCCTTTGTTGCCACATA
TRV2:NbREV3	This paper	F: CGGAATTCCGAAACGCCCCTCACATAAAAG<br>R: CGGGATCCCGATAAGGCAGAGCTCCATG
**Oligonucleotides to clone in pGADT7 and pGBKT7**		
AD-SlPOLA2	This paper	F: GCCAGTGAATTCATGGAAGAGGAAATCAAAG<br>R: GCTCGATGGATCCTTATATACGAAGGACTG
BD-C3 (TYLCV)	This paper	F: GAGGCCGAATTCATGGATTCACGCAC<br>R: CAGGTCGACGGATCCTTAATAAAATTTATATTTTATATCATG
**Oligonucleotides for qPCR and qRT-PCR**		
25S ribosomal DNA interspacer (ITS)	Rosas-Diaz et al., 2018	F: ATAACCGCATCAGGTCTCCA<br>R: CCGAAGTTACGGATCCATTT
NbACTIN	Maimbo et al., 2010	F: CGGAATCCACGAGACTACATAC<br>R: GGGAAGCCAAGATAGAGC
Rep (TYLCV)	Maimbo et al., 2010	F: TGAGAACGTCGTGTCTTCCG<br>R: TGACGTTGTACCACGCATCA
C2 (TYLCV)	Wang et al., 2017b	F: ACCTTCGTCACCCTCTACGA<br>R: AAACGCCATTCTCTGCCTGA
C3 (TYLCV)	This paper	F: TGGACGACATTACAGCCTCA<br>R: ACAATACATGATCAACTGCTCTGA
C4 (TYLCV)	Rosas-Diaz et al., 2018	F: TGCTGACCTCCTCTAGCTGA<br>R: ATCCGAACATTCAGGCAGCT
CP (TYLCV)	Wang et al., 2017b	F: TGGAAGCAGCCCAATGGATT<br>R: GTTCTCGTACTTGGCTGCCT
V2 (TYLCV)	Wang et al., 2017b	F: ATCTGTTGTAAGGGCCCGTG<br>R: CTTTCGGTACATGGGCCTGT
IR (TYLCV)	This paper	F: GGCATGTTGAAATGAATCGG<br>R: GGTCCCACATATTGCAAGAC
Rep (BCTV)	This paper	F: AATGCAAGAATGGGCTGATGC<br>R: GCCCACATAGTCTTCCCTGTT
NbPOLA2 (NbPOLA2.1)	This paper	F: TGGAGAAAGAAGTGAAGGGGAAG<br>R: TGACAAATCACAGCTTCCGTG
NbPOLD2	This paper	F: TTGATTTTCTCCTTTTCCAGATTGC<br>R: TTTGCTGGATCACTGGACCC
NbPOLE2	This paper	F: ACTATTCCGCCAAGATTTGCT

		R: TGGTTTCTTCTGTTGAGGGAGG
NbPOLA1 (NbPOLA1.1/1.2)	This paper	F: TAGTTGCTAAGCGCCGTGAA<br>R: CGGCAACTGACCAATCCTCT
NbPOLA2.2	This paper	F: GCGTTGAGCATTCTGGAGGT<br>R: GAAGGTGCAGGATTTTCGGTAG
NbPOLA3.1/3.2	This paper	F: GCTCCAAACCCCTTAAGCCA<br>R: GAAACCACCTGGCTCATTGC
NbPOLA4.1	This paper	F: AAATTCCCATTTATGCATCCAGC<br>R: ACCTGGAAATGGCGGGAAAA
NbPOLA4.2	This paper	F: CCGAAGCAAATCATACGGCT<br>R: TGTACAAAGAAGGCGACGATG
NbPOLD1	This paper	F: AAGCTTTTTGGGAGGCTATGGA<br>R: TCATGAACACATCCTGATGGAGA
NbPOLE1	This paper	F: GCTGATCCAGAAGTGGAGGG<br>R: CATCGGACAAGGGGCACTC
**Oligonucleotides for two-step anchored qPCR**		
TAG	Rodriguez-Negrete et al., 2014	AGTTTAAGAACCCTTCCCGC
OCS	Rodriguez-Negrete et al., 2014	GGACTTTACATGGGCCTTCAC
OVS	Rodriguez-Negrete et al., 2014	GAAGGCTGAACTTCGACAGC
OCS-TAG	Rodriguez-Negrete et al., 2014	AGTTTAAGAACCCTTCCCGCGGACTTTACATGGGCCTTCAC
OVS-TAG	Rodriguez-Negrete et al., 2014	AGTTTAAGAACCCTTCCCGCGAAGGCTGAACTTCGACAGC
**Oligonucleotides for generating TYLCV-C3mut**		
TYLCV-C3mut	This paper	F: GGGGATTGTTTATCTCCTAAATAAAAACGCCATTCTC<br>R: GAGAATGGCGTTTTTATTTAGGAGATAAACAATCCCC

Supplementary table 3. Primers used in this work.

SUPPLEMENTARY REFERENCES

Briddon, R.W., Watts, J., Markham, P.G., and Stanley, J. (1989). The coat protein of beet curly top virus is essential for infectivity. *Virology 172*, 628-633.

Maimbo, M., Ohnishi, K., Hikichi, Y., Yoshioka, H., and Kiba, A. (2010). S-glycoprotein-like protein regulates defense responses in Nicotiana plants against Ralstonia solanacearum. *Plant Physiology 152*, 2023-2035.

Medina-Puche, L., Huang, T., Dogra, V., Wu, M., Rosas-Diaz, T., Wang, L., Ding, X., Zhang, D., Fu, X., Kim, C., and Lozano-Duran, R. (2020). A defence pathway linking plasma membrane and chloroplasts and co-opted by pathogens. *Cell 182*, 1109-1124.

Rodriguez-Negrete, E.A., Sanchez-Campos, S., Canizares, M.C., Navas-Castillo, J., Moriones, E., Bejarano, E.R., and Grande-Perez, A. (2014). A sensitive method for the quantification of virion-sense and complementary-sense DNA strands of circular single-stranded DNA viruses. *Scientific Reports 4*, 6438.

Rosas-Diaz, T., Zhang, D., Fan, P., Wang, L., Ding, X., Jiang, Y., Jimenez-Gongora, T., Medina-Puche, L., Zhao, X., Feng, Z., *et al.* (2018). A virus-targeted plant receptor-like kinase promotes cell-to-cell spread of RNAi. *Proceedings of the National Academy of Sciences of the United States of America 115*, 1388-1393.

Wang, L., Ding, X., Xiao, J., Jimenez-Gomicronngora, T., Liu, R., and Lozano-Duran, R. (2017a). Inference of a Geminivirus-Host Protein-Protein Interaction Network through Affinity Purification and Mass Spectrometry Analysis. *Viruses 9*.

Wang, L., Tan, H., Wu, M., Jimenez-Gongora, T., Tan, L., and Lozano-Duran, R. (2017b). Dynamic Virus-Dependent Subnuclear Localization of the Capsid Protein from a Geminivirus. *Frontiers in Plant Science 8*, 2165.

Wu, M., Ding, X., Fu, X. & Lozano-Duran, R. Transcriptional reprogramming caused by the geminivirus Tomato yellow leaf curl virus in local or systemic infections in Nicotiana benthamiana. *BMC Genomics* 20, 542 (2019).
